# Laminar flow inhibits the Hippo/YAP pathway via autophagy and SIRT1-mediated deacetylation against atherosclerosis

**DOI:** 10.1038/s41419-020-2343-1

**Published:** 2020-02-21

**Authors:** Ping Yuan, Qiongying Hu, Xuemei He, Yang Long, Xueqin Song, Fei Wu, Yanzheng He, Xiangyu Zhou

**Affiliations:** 1 Department of Neurology, the Affiliated Hospital of Southwest Medical University, Luzhou, 646000 China; 2 Department of Laboratory Medicine, Hospital of Chengdu University of Traditional Chinese Medicine, Chengdu, 610072 China; 3 State Key Laboratory of Biotherapy, Sichuan University, Chengdu, 610072 China; 4 Medical Research Center, the Affiliated Hospital of Southwest Medical University, Luzhou, 646000 China; 5 Department of Thyroid and Vascular Surgery, the Affiliated Hospital of Southwest Medical University, Luzhou, 646000 China

**Keywords:** Molecular biology, Atherosclerosis

## Abstract

Atherosclerosis is a multifactorial disease of the vasculature, and shear stress is a crucial regulator of its process. Disturbed flow promotes atherosclerotic effects, while laminar flow has a protective action on the endothelium. Hippo/YAP is a major cascade that senses various mechanical cues and mediates the expression of pro-inflammatory genes. However, the mechanism modulating the transcription factor YAP in response to different patterns of blood flow remains unclear. In this study, we provide evidence that shear stress modulates YAP activity via autophagy in endothelial cells. Laminar flow promoted the expression of the autophagic markers BECLIN 1 and LC3II/LC3I. Autophagy blockade using a chemical inhibitor repressed YAP degradation under laminar flow. Conversely, the induction of autophagy under disturbed flow partially antagonized the nuclear import and transcriptional activation of YAP. In parallel, laminar flow led to the increased expression of SIRT1 protein, a NAD^+^-dependent deacetylase. Further investigation showed that SIRT1-mediated YAP deacetylation. The forced expression of SIRT1 under disturbed flow effectively attenuated YAP activation and nuclear accumulation, thereby downregulating the expression of pro-inflammatory genes. In atheroprone vessels of mice receiving rapamycin to induce autophagy, the enhanced expression of SIRT1 was observed together with YAP repression. Altogether, these results show that endothelial autophagy and SIRT1 expression induced by laminar flow contribute to the inhibition of Hippo/YAP signaling and interrupt atherosclerotic plaque formation.

## Introduction

Atherosclerosis, as a major vascular disease, is a progressive illness caused by inflammatory events in endothelial cells (ECs) of large or medium arteries^1^. The EC monolayer, located at the interface between the circulating blood and vessel wall, is directly exposed to blood flow and associated shear stress^2^. Shear stress plays crucial roles in the maintenance and disturbance of vascular homeostasis, and different flows greatly modify endothelial phenotypes and determine the uneven distribution of atherosclerotic lesions^3^. In straight sections of the arterial tree, steady unidirectional laminar flow (UF) induces endothelial protective effects, such as anti-oxidation, anti-inflammation, and anti-atherogenesis; while in branches and curved sections, disturbed flow (DF) evokes pro-atherogenic responses, such as oxidative stress, inflammation, and atherogenesis^4^. Local flow around the atherosclerotic micro-environment defines the endothelial phenotype, which leads to the initiation and development of plaques^5^.

DF and UF modulate different aspects of ECs via mechanosensitive transcription factors, such as KLF2/4, HIF-1α, YAP/TAZ/TEAD, NF-κB, NRF2, and AP-1^4^. Accumulating studies have indicated that YAP/TAZ/TEAD signaling represents a promising therapeutic target for the prevention and treatment of atherosclerosis^4^. Yes-associated protein (YAP) and transcriptional co-activator with PDZ-binding motif (TAZ) are effectors of the Hippo pathway, which is important in the control of cell function and consists of a series of serine/threonine kinases, including MST1/2, SAV1, MOB1, and LATS1/2. MST1/2 and LATS1/2 kinases have been found to control YAP activity via the phosphorylation of Ser127 in YAP^6^. When Hippo signaling is activated, the upstream kinases MST1/2 are activated, which in turn phosphorylate and activate LATS1/2; LATS1/2 then phosphorylates YAP and TAZ; phosphorylation of YAP at Ser127 facilitates the association of YAP with 14-3-3 protein, functionally retaining YAP in the cytoplasm and promoting its degradation via the ubiquitin-proteasome system; when the Hippo pathway is deactivated, YAP and TAZ are dephosphorylated and imported into the nucleus, where they form active transcriptional complexes with DNA-binding transcription factors including TEA-domain (TEAD) transcription factors^7^. These transcriptional complexes induce the expression of a wide range of genes (e.g., cytoskeletal regulators, cellular organelles, components of cell–cell junctions, inflammatory molecules, and secreted proteins) that are involved in cell growth, apoptosis, migration, and communication^8,9^. In addition, YAP is also an autophagy substrate^10^. Aurora A can block autophagy to maintain YAP stability and increase YAP protein abundance^11^.

Recently, YAP was identified as a crucial factor in atherosclerosis. In human umbilical vein endothelial cells (HUVECs), the phosphorylation of YAP at Ser127 was reduced under DF, which was accompanied by the increased expression of YAP target genes^12^, suggesting the activation of YAP under DF. Increased nuclear YAP and decreased phosphorylated YAP were also found in atherosclerosis-prone regions of arteries in mice^13^. Depletion of *Yap* in endothelial cells (ECs) impedes plaque formation in apolipoprotein E-deficient (*ApoE*^*−/−*^) mice^14^. Mechanistic studies have revealed that UF favors the interaction of integrin with Gα13 and thereby causes RhoA inhibition, further triggering YAP phosphorylation and repression^12^. DF leads to the continuous activation and nuclear translocation of YAP in ECs, which is dependent on integrin α5β1 activation. The activation of integrin α5β1 induces the phosphorylation of YAP at Tyr357 via c-Abl tyrosine kinase, which usually colocalizes with integrin α5 and is activated in cells treated with fibronectin or fluid shear stress^15^. Bosutinib, a tyrosine kinase inhibitor, greatly inhibits the phosphorylation of YAP at Tyr357 and the generation of atherosclerosis in *ApoE*^−/−^ mice^15^. Taken together, these results indicate that the YAP pathway holds promise as a novel drug target against atherosclerosis.

However, it is unclear how laminar flow limits the activation of Hippo/YAP signaling to hamper the formation of atherosclerotic lesions. Autophagy is a protective and adaptive mechanism for the maintenance of vascular homeostasis. Herein, our results suggested that UF-induced endothelial autophagy facilitates YAP degradation to repress the Hippo pathway in vascular ECs. Conversely, inhibition of autophagy using a chemical inhibitor enhanced the stability of YAP. Meanwhile, UF also induced SIRT1 expression and SIRT1-mediated YAP deacetylation to promote the nuclear export of YAP and subsequent degradation via autophagy. SIRT1 dysfunction and YAP activation were found in areas of the vasculature that are normally prone to atherosclerosis. Rapamycin attenuated atherosclerosis induced by dietary cholesterol in *ApoE*^−/−^ mice via upregulating SIRT1 and inactivating YAP. Therefore, different forms of extracellular fluid shear stress can affect the activity of the YAP pathway via autophagy and SIRT1-mediated deacetylation. Our findings provide a novel insight into the prevention and treatment of atherosclerosis.

## Results

### Shear flow regulates YAP activity

**Fig. 1:**
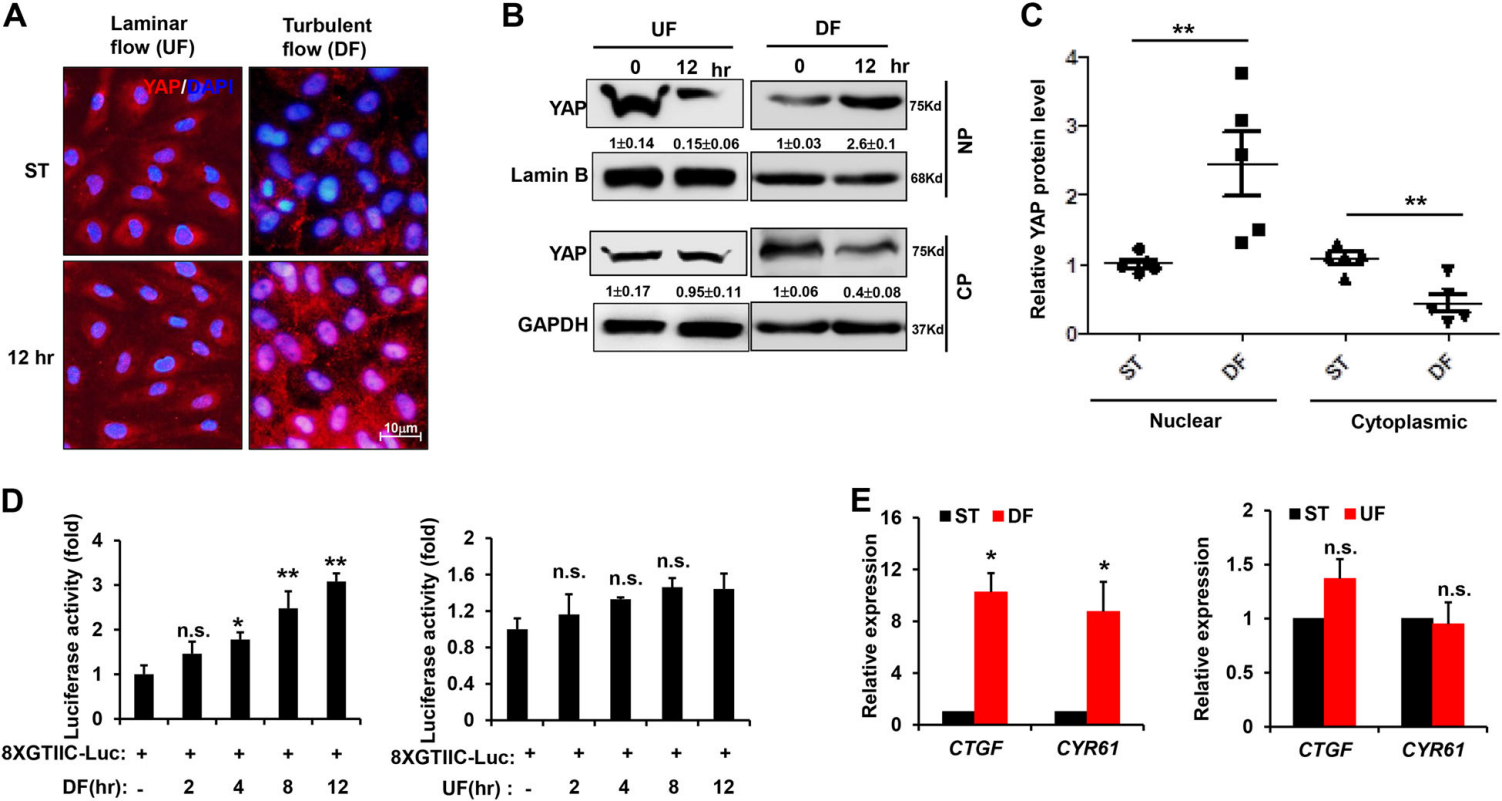
Shear flow regulates YAP phosphorylation, subcellular localization, and downstream gene expression. **a** HUVECs were exposed to UF (12 dyn/cm^2^) or DF (0.5 ± 6 dyn/cm^2^, 1 Hz) for the indicated times. Cells with static treatment (ST) were a control. After treatment, cells were underwent immunofluorescence staining with YAP (red) and DAPI (blue). **b** Western blot analysis showed YAP expression and YAP subcellular distribution (NP, nuclear protein; CP, cytoplasmic protein) under UF and DF flow condition in HUVEC cells. Numbers under the blots were mean ± SD of three biologically independent experiments, and the first lane (0 h) was served as relative control. **c** Analyzed the relative total YAP protein level in nuclear and cytoplasm using gray density analysis for five independent experiments. GAPDH and Lamin B were used as a loading control for cytoplasmic and nuclear fractions. **d** HUVECs were transfected with 8 × GTIIC reporter and renilla-luc plasmids and were subjected to shear flow 48 h post transfection. Then the relative firefly/renilla luciferase activity was determined. **e** The expression of YAP target genes *CTGF* and *CYR61* was determined by real-time PCR. Data represent mean ± SD from three independent experiments (**P < 0.01, *P < 0.05, vs. the control).

To investigate the effects of shear flow on YAP activity in endothelial cell, we used a HUVEC cell line which is reliable and widely used in studying the mechanism of arteriosclerosis, because HUVEC cells are characterized by unlimited growth while maintaining endothelial cell characteristics^16^. HUVECs were subjected to UF (12 dyn/cm^2^) or DF (0.5 ± 6 dyn/cm^2^, 1 Hz) for 12 h and we found that DF treatment drove the cytoplasm-to-nucleus translocation of YAP, while no significant change was observed in HUVECs subjected to UF as shown by immunofluorescence assay (Fig. 1a). Western blot analysis further confirmed the UF treatment decreased the total abundance of YAP protein while DF stimulated YAP amount and increased total expression and nuclear localization of YAP compared with static cells (Fig. 1b). Quantification of the western blots showed that cyto-nuclear translocation of YAP under DF stimulation was significant (*P* < 0.001) (Fig. 1c). Subsequently, HUVECs transfected with a YAP/TAZ-responsive luciferase (8 × GTIIC-luc) reporter under the control of eight tandem copies of the TEAD-binding site (ACATTCCA) were subjected to UF or DF. Only DF exposure led to the increased transcriptional activity of YAP (Fig. 1d). Congruently, DF effectively enhanced the expression of the YAP/TAZ target genes *CTGF* and *CYR61* (Fig. 1e). Thus, YAP activity is greatly affected by shear flow and UF limits YAP activation.

### Shear flow affects YAP degradation via autophagy

**Fig. 2:**
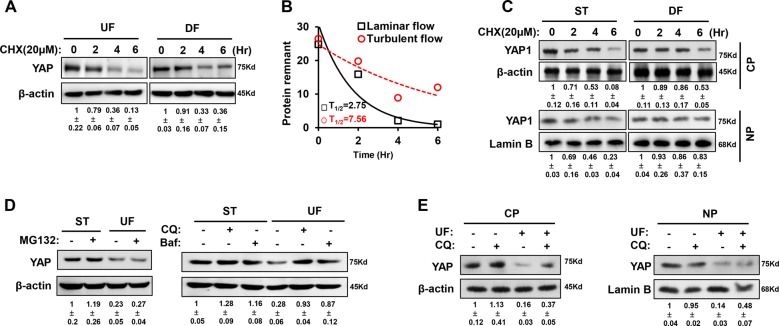
Different degradation levels of YAP protein in response to shear flow. **a** HUVECs were treated with UF or DF for the indicated times in the presence of 20 μM CHX. The half-life of endogenous YAP protein was determined by western blot. **b** Calculation of half-life (T1/2) of the YAP protein in laminar flow and disturbed flow. **c** Cytoplasmic and nuclear YAP proteins from HUVECs treated with DF were extracted sequentially for western blot assay to detect the protein degradation. NP and CP represent nuclear and cytosolic proteins, respectively. **d** HUVECs were treated by UF in the presence of the proteasome inhibitor MG132 (10 μM) or autophagy inhibitors bafilomycin A1 (Baf, 1 μM) and chloroquine (CQ, 10 μM) for the indicated times, and then western blot analysis was employed to assess YAP abundance. **e** After UF treatment combined with 10 μM of chloroquine, cytoplasmic and nuclear fractions from HUVECs were extracted for western blot analysis to assess YAP amount. Representative images from at least three independent experiments are shown. Numbers under the blots were mean ± SD of three biologically independent experiments, and the first lane was served as relative control.

As UF downregulated the total abundance of YAP, we subsequently wanted to ascertain how YAP is inactivated under UF. A previous study revealed that YAP can be inactivated by its cytoplasmic sequestration and degraded by the ubiquitin-proteasome system or autophagy. Using a cycloheximide (CHX) chase experiment, we compared the half-life of YAP protein under UF or DF. As shown in Fig. 2a, the half-life of YAP protein was increased under DF from 2.75 to 7.56 h (Fig. 2b). A further experiment examining YAP protein in different intracellular compartments showed that DF mainly prolonged the half-life of cytoplasmic YAP; however, nuclear YAP was more stable than cytoplasmic YAP and was less affected by shear flow (Fig. 2c).

To further determine the main pathway for YAP degradation, HUVECs were treated with the proteasome inhibitor MG132 or autophagy inhibitors (bafilomycin A1 and chloroquine) and western blot analysis was performed to assess YAP abundance under UF. Autophagy inhibitors significantly reversed the downregulation of YAP induced by UF treatment for 12 h (Fig. 2d). Meanwhile, western blot analysis suggested that treatment with chloroquine mainly enhanced the levels of cytoplasmic YAP rather than nuclear YAP (Fig. 2e). These results indicate that shear flow may affect YAP stability via autophagy.

### Laminar flow promotes autophagy against atherosclerosis

**Fig. 3:**
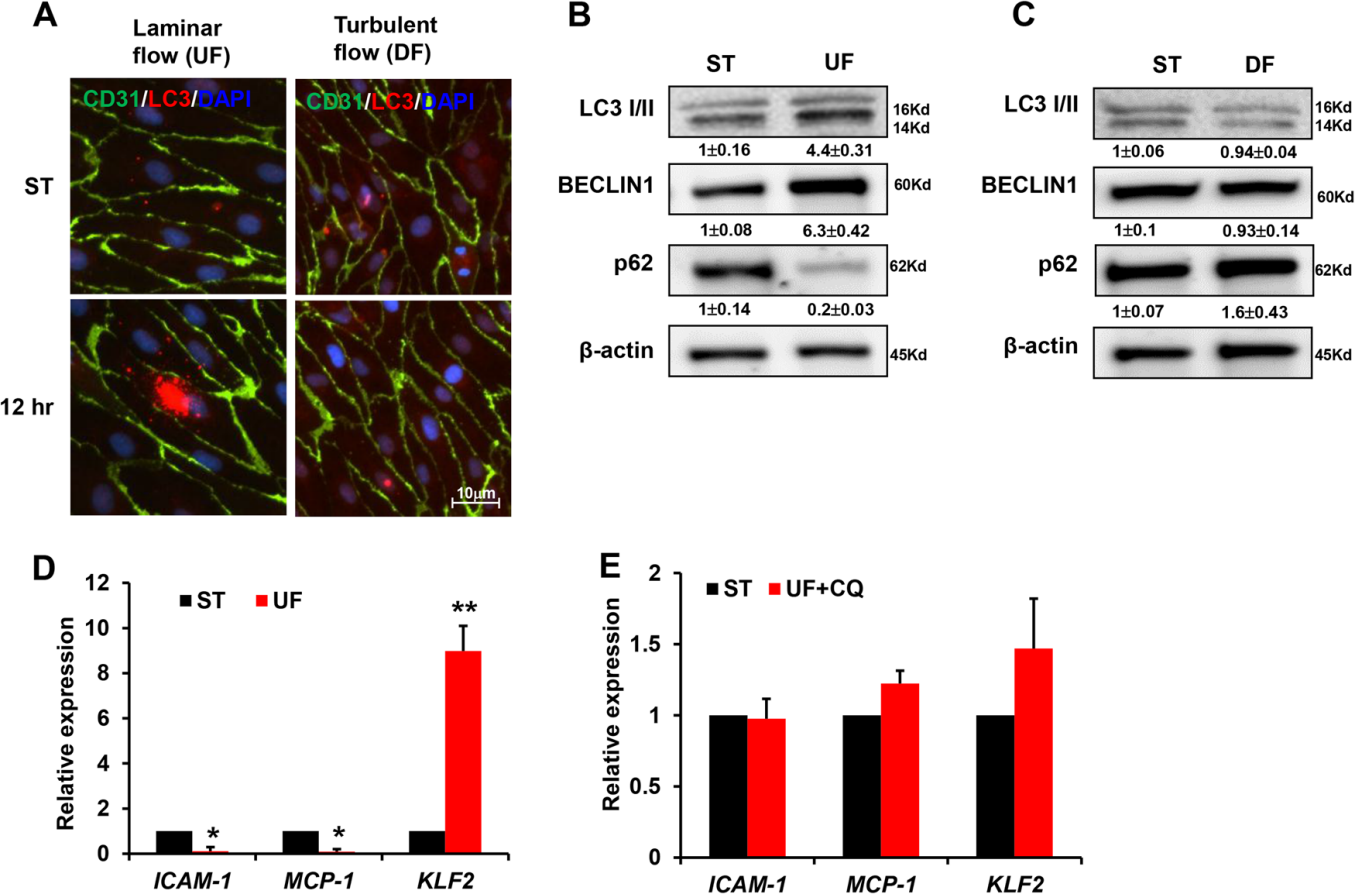
Laminar flow induces autophagy. **a** HUVECs were exposed to shear stress for 12 h and then subjected to immunofluorescence staining. Endothelial cells were labeled with anti-CD31 antibody. LC3 puncta indicated that UF significantly increased the number of autophagosomes in comparison with DF. Representative images from at least three independent experiments are shown. **b**, **c** Western blot analysis was performed to show the expression of BECLIN, LC3II/ LC3I, and p62 under UF or DF. Numbers under the blots were mean ± SD of three biologically independent experiments, and the first lane (ST) was served as relative control. HUVEC cells were treated with UF (**d**) or (**e**) with autophagy inhibitors, real-time PCR was performed to determine the expression of *ICAM1*, *MCP-1*, and *KLF2*. Data represent mean ± SD of three independent experiments (**P < 0.01, *P < 0.05, vs. the control).

We subsequently examined autophagy levels in HUVECs by monitoring LC3 puncta with immunofluorescence. LC3 staining was relatively weaker in CD31-positive ECs treated with DF compared with those exposed to physiological UF (Fig. 3a). Western blot analysis also confirmed that UF treatment for 12 h promoted the expression of the autophagic marker BECLIN 1 and the conversion of LC3-I into LC3-II, whereas the levels of the autophagic substrate p62 decreased (Fig. 3b). In contrast, DF failed to activate autophagy (Fig. 3c). Furthermore, UF downregulated the expression of the pro-inflammatory genes *ICAM1* and *MCP-1*, while it increased the expression of the anti-inflammatory gene *KLF2* (Fig. 3d). However, the autophagy inhibitor chloroquine was partially antagonistic to the protective effects of UF, since treatment of chloroquine under UF condition reversed the downregulation of *ICAM1* and *MCP-1*, but enervated the upregulation of *KLF2* remarkably (Fig. 3e). Collectively, the results of the present study indicate that UF increases the level of autophagy and thereby protects ECs against atherosclerosis by preventing inflammation.

### Autophagy modulates the transcriptional program of YAP

**Fig. 4:**
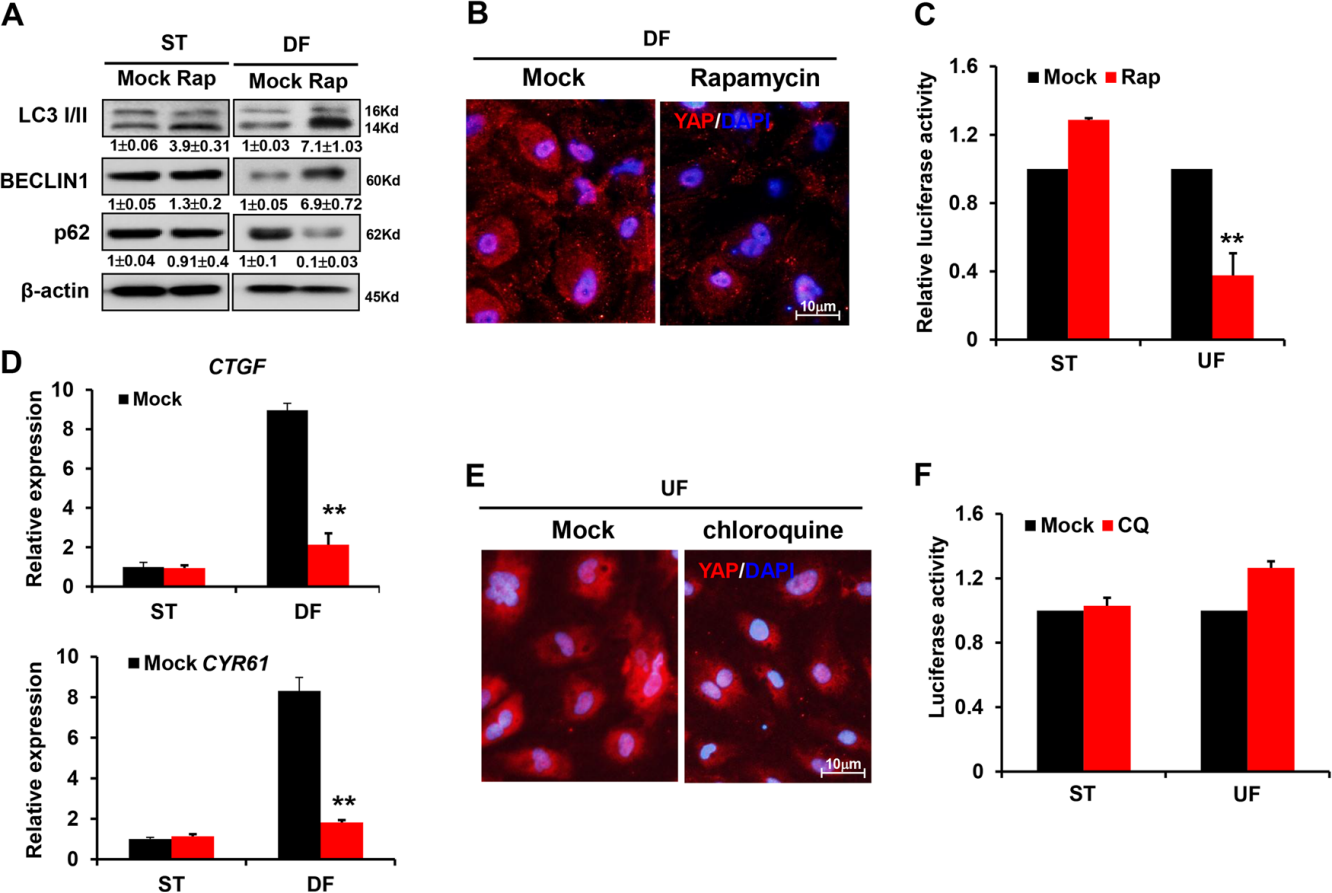
Autophagy modulates YAP. **a** After DF treatment together with 100 nM rapamycin administration for 12 h, western blot analysis was performed to show the expression of BECLIN 1, LC3II/ LC3I, and p62. Numbers under the blots were mean ± SD of three biologically independent experiments, and the first lane (Mock) was served as relative control. **b** Immunofluorescence staining showed the total abundance and nuclear enrichment of YAP protein in the presence of 100 nM rapamycin for 12 h. **c** Luciferase assay showed the activity of 8 × GTIIC reporter in HUVECs exposed to 100 nM of rapamycin treatment under DF for 12 h, and **d** real-time PCR showed the expression levels of YAP downstream genes *CTGF* and *CYR61*. **e** Immunofluorescence assay showed that chloroquine treatment enhanced YAP staining in the cytoplasm under UF. **f** Luciferase assay showed the activity of 8 × GTIIC reporter in HUVECs exposed to chloroquine under UF. Data represent mean ± SD of three independent experiments (**P < 0.01, *P < 0.05, vs. the control).

To ascertain whether autophagy inhibits the development of atherosclerosis by manipulating YAP, we treated HUVECs with a pharmacological inhibitor or agonist of autophagy under different patterns of shear flow. Rapamycin, an inhibitor of mTOR, remarkably induced autophagy in HUVECs exposed to DF (Fig. 4a). Simultaneously, rapamycin decreased the total abundance and nuclear enrichment of YAP protein under DF (Fig. 4b). As a consequence, rapamycin treatment decreased the transcriptional activity of the 8 × GTIIC-luc reporter, demonstrating the downregulation of YAP function (Fig. 4c). The expression levels of the YAP downstream genes *CTGF* and *CYR61* were reduced after exposure to rapamycin (Fig. 4d).

By comparison, the autophagy inhibitor chloroquine interrupted YAP degradation under UF and enhanced YAP staining in the cytoplasm, as evidenced by immunofluorescence (Fig. 4e). Consistently, the transcriptional activity of YAP under UF was not significantly elevated by chloroquine (Fig. 4f). Therefore, autophagy inhibition is required for the cytoplasmic enrichment of YAP, where YAP exists in an inactive form. It is thus likely that other stimulatory signals are integrated to drive the nuclear import and transcriptional activation of YAP.

### UF-induced SIRT1 deacetylates YAP

**Fig. 5:**
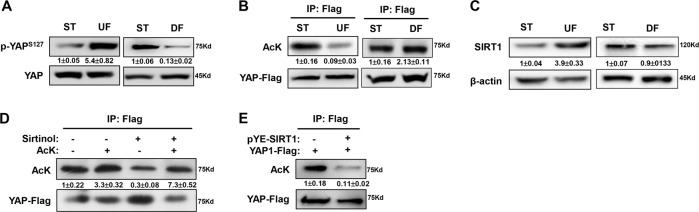
UF upregulated SIRT1 to deacetylate YAP. **a** Immunoblotting showed YAP phosphorylation at S127 after HUVECs were exposed to UF or DF. **b** Cell lysates from HUVECs treated by DF and UF shear flow were subjected to immunoprecipitation with anti-YAP antibody, and bound proteins were analyzed using specific antibody against acetylated-lysine (AcK), the YAP-flag was used as endogenous control. **c** Western blot showed the expression of SIRT1 under UF and DF. **d** Immunoprecipitation assay showed the acetylation level of YAP protein from HUVECs treated by UF together with 10 μM sirtinol. **e** 293T cells were transfected with FLAG-SIRT1 and pcDNA4/HisMaxB-YAP1 plasmids. Forty-eight hours later, immunoprecipitation assay was performed to show the acetylation status of YAP. Representative images from at least three independent experiments are shown. Numbers under the blots were mean ± SD of three biologically independent experiments, and the first lane (ST) was served as relative control.

Post-translational modifications, such as phosphorylation, acetylation, and ubiquitination, define the transcriptional activation of YAP. In this study, we found that DF repressed the phosphorylation of YAP at Ser127, thereby activating YAP; UF treatment led to increased YAP phosphorylation at Ser127 (Fig. 5a). Meanwhile, we performed immunoprecipitation and western blot experiments to detect the acetylation of YAP protein under the DF and UF stimulations. The acetylation level of YAP was decreased under UF, indicating a positive association between acetylation and YAP activation, but no significant change was detected under DF stimulation, indicating other regulating mechanisms may be involved (Fig. 5b). Therefore, we speculated that UF might increase the expression of deacetylases to limit YAP activity.

Previous studies revealed that UF induces SIRT1 to favor autophagy, modulate homeostasis, and antagonize atherosclerosis. SIRT1 is a NAD^+^-dependent deacetylase targeting various substrates. Herein, our results also confirmed that UF treatment for 12 h upregulated SIRT1 expression, but no significant change was observed under DF stimulation (Fig. 5c). Treatment with the SIRT1 inhibitor sirtinol alone was sufficient to block UF-induced YAP deacetylation (Fig. 5d). Furthermore, the transient overexpression of plasmids encoding SIRT1 and YAP in HEK293T cells indicated that SIRT1 could downregulate the acetylation level of YAP protein (Fig. 5e). Taken together, our results indicate that UF can induce SIRT1 to inhibit YAP acetylation, thereby constituting a putative mechanism by which UF modulates YAP activity.

### SIRT1 regulates the nuclear export and degradation of YAP

**Fig. 6:**
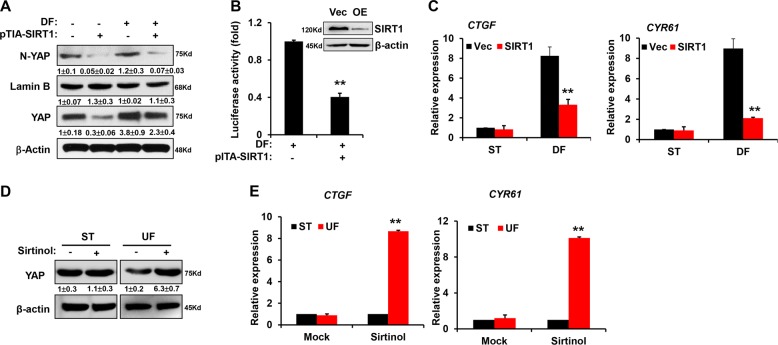
SIRT1 promotes the nuclear export and degradation of YAP under UF. **a** HUVECs were infected with lentivirus overexpressing SIRT1 for 72 h and the total abundance and nuclear enrichment of YAP protein in the presence of SIRT1 overexpression was detected using western blot. N-YAP represented nuclear YAP. Numbers under the blots were mean ± SD of three biologically independent experiments, and the first lane was served as relative control. **b** Luciferase assay showed the activity of 8 × GTIIC reporter in SIRT1-overexpressing HUVECs exposed to DF. Upper panel, western blot to confirm the SIRT1 level after overexpression. **c** Real-time PCR showed the expression levels of YAP downstream genes *CTGF* and *CYR61* in SIRT1-overexpressing HUVECs. **d** HUVECs were treated with 10 μM sirtinol and exposed to UF. Then, western blot showed the total abundance of YAP protein under UF. **e** Real-time PCR showed the expression levels of YAP downstream genes *CTGF* and *CYR61* under the treatment of 10 μM sirtinol together with UF.

Since YAP might be a downstream substrate of SIRT1, we examined the effects of SIRT1 on YAP localization and activity. We overexpressed SIRT1 in HUVECs by using a lentivirus harboring a SIRT1-flag gene fragment. SIRT1 overexpression significantly inhibited the DF-induced nuclear accumulation of YAP, and the total abundance of YAP protein was also decreased in the presence of SIRT1 overexpression (Fig. 6a). These findings suggest that YAP proteins exported from the nucleus are susceptible to degradation. Coincidentally, YAP/TAZ-responsive luciferase reporter activity under DF was also reduced by overexpressing SIRT1 (Fig. 6b). SIRT1 overexpression also decreased the expression of the YAP downstream genes *CTGF* and *CYR61* under DF (Fig. 6c). By comparison, treatment with the SIRT1 inhibitor sirtinol alone upregulated YAP abundance under UF and increased the expression of *CTGF* and *CYR61* (Fig. 6d, e). Taken together, these results indicate the atheroprotective effect of SIRT1 by facilitating the nuclear export of YAP.

### Rapamycin attenuates atherosclerotic plaque progression in mice via YAP inhibition

**Fig. 7:**
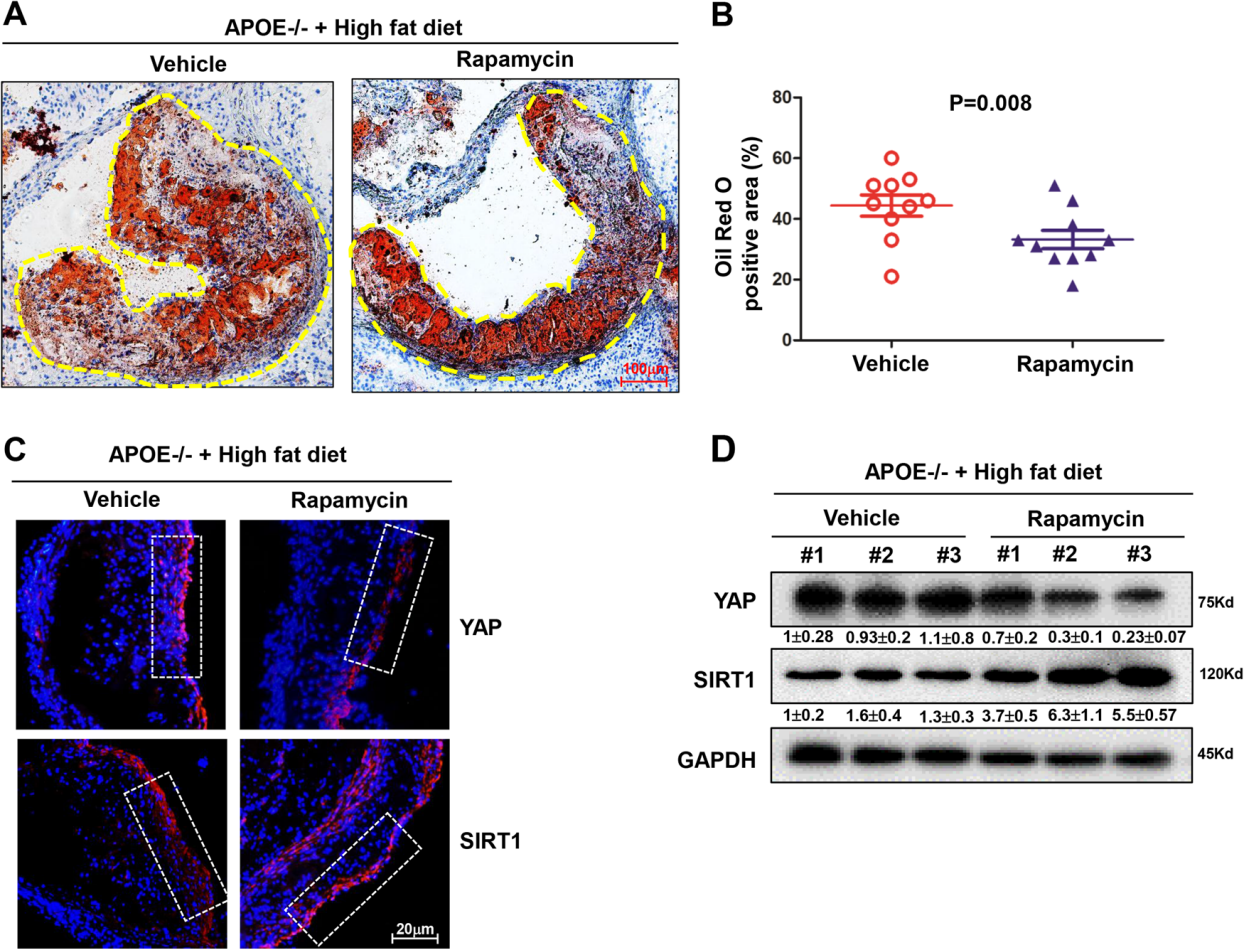
Rapamycin attenuates atherosclerotic progression in mice. **a** Oil Red O staining of mice aortas in the *ApoE*^−/−^ mice fed a diet supplemented with cholesterol (Vehicle group) or with cholesterol plus Rapamycin (Rapamycin) for 8 weeks, and yellow dashed line indicates the size of plaque area as a percentage of total area. **b** Quantification of plaque size, Oil Red O-positive area in Vehicle group (*n* = 10) and rapamycin group (*n* = 10). Data are mean ± SEM. **c** Immunofluorescence staining for YAP and SIRT1 proteins in the atherosclerotic vessels from the vehicle group and rapamycin group. Nuclei are counterstained with DAPI (blue). White frame indicates endothelia membrane. Representative images are shown, *n* = 10. **d** Western blotting showed the total YAP protein level and SIRT1 level in tissues from vehicle group and rapamycin group. Three samples of each group were shown. Numbers under the blots were mean ± SD of three biologically independent experiments, and the first lane was served as relative control.

A further experiment was designed to test whether autophagy inhibits atherosclerotic plaque progression by modulating SIRT1 and YAP. *ApoE*^−/−^ mice were fed either a diet supplemented with cholesterol (Vehicle group) or with cholesterol plus rapamycin (Rapamycin group). At 8 weeks, quantitative analyses of plaque area were performed. In the Rapamycin group, plaque area was significantly reduced compared with the Vehicle group (Fig. 7a), and the difference was significant between both groups (Fig. 7b).

We next determined the expression of SIRT1 and YAP in the atheroprone regions of *ApoE*^−/−^ mouse aortas. In the inner curvature and bifurcation of the aortic arch exposed to DF, YAP was expressed at higher levels in the Vehicle group when detected using an immunofluorescence assay (Fig. 7c). Meanwhile, when these tissues were separated for western blot assay, we also confirmed that rapamycin treatment effectively decreased YAP expression and simultaneously augmented SIRT1 expression (Fig. 7d). Therefore, these results indicate that rapamycin can inhibit diet-induced atherosclerosis in *ApoE*^−/−^ mice by activating autophagy to deplete YAP.

## Discussion

The transcriptional activator YAP is a potential therapeutic target against atherosclerosis. YAP can act as a sensor and signal amplifier of mechanical stimuli including matrix stiffness, stretching, and cell density. The results of this study revealed that UF induces autophagy to perturb YAP activation, while DF can relieve the autophagic inhibition of YAP.

YAP can respond to mechanical stress and trigger diverse biomechanical effects. The repression of YAP in response to laminar flow is required for the maintenance of vessel homeostasis in endothelial cells. Previous study showed that the atheroprotective laminar flow represses YAP activity via the integrin–Gα_13_–RhoA pathway in endothelial cells^12^. In contrast, YAP can be activated by disturbed flow. YAP activation induced by atheroprone-disturbed flow enhances JNK activity to favor inflammation and atherogenesis. Further investigation indicated that oscillatory shear stress induces YAP nuclear translocation via the activation of integrin α5β1 and its downstream kinase c-Abl. c-Abl phosphorylates YAP at Y357 to evoke endothelial atherogenic responses to disturbed flow^15^. In addition, osmotic stress has also recently been shown to stimulate Nemo-like kinase (NLK) and mediate the phosphorylation of YAP at S128, then enhancing nuclear YAP amount^17^. Understanding the context-specific mechanisms regulating YAP activity is important for potential interception of atherosclerosis. Herein, we provided evidence that autophagy has a role in the inactivation and instability of YAP under UF. Increasing data indicate that mechanostimulation can affect autophagy. A study revealed that shear stress transduced by the primary cilium can activate canonical autophagy in kidney epithelial cells^18^. Another study reported that shear force can be sensed by actin-rich microvilli in intestinal epithelial monolayers and induce a noncanonical autophagic flux controlled by autophagy components including ATG5 and LC3, but not BECLIN 1^19^. Hemodynamic shear stress has been found to promote autophagy as a pro-survival mechanism in HeLa cells through lipid raft-mediated mechanotransduction^20^. Shear stress can affect autophagy in ECs; however, the underlying mechanisms and impact of shear stress on autophagy in ECs are not entirely understood. Multiple studies have provided evidence that shear stress induced by UF, but not by oscillatory or low-magnitude flow, favors autophagy^21–23^. Indeed, a previous study revealed that YAP is also an autophagy substrate^10^. Aurora A can inhibit cell autophagy to enhance YAP protein stability independently of the Hippo pathway and proteasome pathway^11^. Herein, our results indicated that UF-induced endothelial autophagy facilitates YAP degradation to inhibit the Hippo pathway. Conversely, pharmacological inhibition of autophagy enhanced the stability of YAP, and then increased the expression of inflammatory genes such as *CTGF* and *CYR61*. Intriguingly, autophagy inhibition under UF only enhanced YAP staining in the cytoplasm, where YAP is present in an inactive form. Seemingly, there exist other mechanisms that further activate YAP and evoke its nuclear import. Thus, YAP is modulated at different levels. For YAP degradation, YAP should first undergo inactivation and nuclear export, and then YAP should be degraded by autophagosomes.

Post-translational modifications, such as phosphorylation, acetylation, and ubiquitination, might contribute to the nucleocytoplasmic shuttling and complete activation of YAP protein^8,9^. The subcellular localization and transcriptional activity of YAP is tightly linked with its phosphorylation status. AMPK has recently been shown to phosphorylate YAP at Ser94 under conditions of energy stress, consequently disrupting the mutual interactions between YAP and TEAD/TEF transcription factors^24,25^. Our investigation showed that SIRT1-mediated YAP deacetylation promoted the nuclear export of YAP and its subsequent degradation. SIRT1 overexpression directly repressed the DF-induced nuclear localization of YAP, thereby downregulating the expression of YAP target genes. Therefore, our data indicate that SIRT1 is an important modulator of the subcellular localization and transcriptional activation of YAP. UF-induced SIRT1 expression was crucial for cell autophagy and YAP repression.

Furthermore, we provided evidence that the oral autophagy inducer rapamycin could effectively hamper the growth of atherosclerotic plaques in *ApoE*^*−/−*^ mice. Rapamycin increased SIRT1 expression and simultaneously repressed YAP in atheroprone areas of vessels. In summary, SIRT1 and autophagy can limit YAP activation to prevent atherosclerosis. The present study and the abovementioned studies together provides a conceptual framework for understanding YAP modulation in different patterns of blood flow, potentially leading to the development of novel therapeutic approaches against the development of atherosclerosis.

## Materials and methods

### Cell culture

Human umbilical vein endothelial cells (HUVECs) were obtained from Type Culture Collection of the Chinese Academy of Sciences (Shanghai, China) and cultured in high-glucose DMEM supplemented with 5% fetal bovine serum (GIBICO) and 1% endothelial cell growth supplement (ECGS), and 100 Units/mL penicillin plus 100 μg/ml streptomycin under a humidified atmosphere containing 5% CO_2_ maintained at 37 °C. For all of the experiments, mycoplasma free HUVECs in passage 3 were used. HEK293T cells were maintained in DMEM supplemented with 10% FBS.

### Reagents and antibodies

Cycloheximide was purchased from Sigma-Aldrich (St Louis, MO, USA). MG132, autophagy inhibitors (bafilomycin A1 and chloroquine), rapamycin, and sirtinol were purchased from Selleck Chemicals (Houston, TX, USA). Antibodies against YAP (#14074), phosphorylated YAP (#13008), BECLIN 1 (#3495), LC3 A/B (#12741), p62 (#8025), SIRT1 (#8469), FLAG tag (#14793), acetylated-lysine (#9441), and β-actin (#3700) were obtained from Cell Signaling Technology (Danvers, MA, USA). CD31 (sc-376764), β-actin (sc-47778), GAPDH (sc-47724), and Lamin B (sc-374015) antibodies were obtained from Santa Cruz Biotechnology (Santa Cruz, CA, USA). Antibody specific for FLAG tag was provided by Sigma-Aldrich (St Louis, MO, USA). FLAG-SIRT1 was kindly provided by Professor Zhiqiang Liu at the Department of Physiology and Pathophysiology, School of Basic Medical Science, Tianjin Medical University. 8 × GTIIC-luc and pcDNA4/HisMaxB-YAP1 plasmids were obtained from Addgene (Watertown, MA, USA).

### Shear stress experiments

The flow experiments were performed as previously described^12^. For flow experiments, confluent monolayers of HUVECs were seeded on μ-slide I^0.4^ Luer (IBIDI, LLC). A parallel-plate flow system (IBIDI, Germany) was used to impose UF and disturbed flow (12 dyn/cm^2^ for UF and 0.5 ± 4 dyn/cm^2^, 1 Hz for disturbed flow). The system was maintained at 37 °C and ventilated with 95% humidified air containing 5% CO_2_.

### Luciferase assay

For detection of the 8 × GTIIC-Luc activity, 0.8 μg of total DNA including 0.2 μg 8 × GTIIC-Luc reporter vector and 1 ng pRL-TK Renila plasmid as internal control were co-transfected into HUVECs with Lipofectamine 3000 (Life Technologies, ThermoFisher Scientific, Waltham, MA, USA). Forty-eight hours post transfection, cells were processed by shear flow. Then, cells were harvested using the Dual-Glo luciferase assay system (Promega, Madison, WI, USA) following manufacturer’s instructions. Luminescence of Firefly and Renilla luciferase activities were quantified using Perkin Elmer EnVision plate reader (Waltham, MA, USA).

### Immunofluorescence staining

After treatment, HUVECs were fixed and washed twice with PBS. After blocking with bovine serum albumin, the cells were incubated with antibodies against LC3, CD31, and YAP. Next, they were incubated with FITC- or TRITC-conjugated secondary antibodies for 2 h at room temperature. DAPI was used to counter stain nucleus of cells. The images of the cells were captured using a fluorescence microscope (IX70, Olympus, Tokyo, Japan).

### Quantitative real-time PCR

Real-time PCR has been descripted previously^26^. Total RNA was isolated with TRIzol reagent (Life Technologies, Carlsbad, CA, USA) and reverse transcribed using random hexamers and the PrimeScript RT Kit from TaKaRa (Dalian, China). Quantitative real-time PCR was performed using SYBR Green real-time Master Mix (Toyobo, Japan) in a Prism 7500 system (Applied Biosystems, Foster City, CA, USA). *GAPDH* was used as the house keeping gene. The 2^−ΔΔCt^ method was used to assess the relative mRNA expression level. Primers used for quantitative real-time PCR were *CTGF:* ATTAGAGCCAACTGCCTGGT, AGGAGGCGTTGTCATTGGTA; *CYR6:* CTGCTCAGCTCCCAGGTC, GGGTCAGTTGTTCCTCCAGT; *MCP1:* CAGCCAGATGCAATCAATGCC, TGGAATCCTGAACCCACTTCT; *ICAM1:* CACAGTCACCTATGGCAACG, CCGGAAAGCTGTAGATGGTC; *KLF2:* CACGCACACAGGTGAGAAG, CACAGATGGCACTGGAATGG; *GAPDH*: CTGGGCTACACTGAGCACC, AAGTGGTCGTTGAGGGCAATG.

### Western blotting (WB)

Western blotting was performed as previously described^26^. Briefly, cells (1 × 10^6^) were lysed on ice in RIPA buffer containing protease inhibitors. The protein content was measured using a BCA Protein Assay Kit (Beyotime Institute of Biotechnology, Beijing, China), and total protein (20 μg) was separated by SDS-PAGE electrophoresis, and transferred to PVDF membranes (Millipore Corporation, USA). The membranes were blocked with 5% non-fat milk for 2 h at room temperature and incubated overnight at 4 °C with antibodies, followed by incubation with specific secondary antibodies. Detection of specific proteins was carried out with an ECL chemiluminescence detection kit (Beyotime Institute of Biotechnology, Beijing, China) according to the manufacturer’s instruction. The western blot images were obtained with an MiniChemi 610 chemiluminescent imager (Sagecreation, Beijing, China). All experiments were performed at least in triplicate, and quantitative analysis of blots were performed using the Fiji based ImageJ software (version 1.51n, National Institute Health, Bethesda, MA, USA).

### Transfection, virus production, and viral transduction

Transient transfections were performed using polyethyleneimine (PEI) transfection reagent (Polysciences) in the OPTI-MEM medium (Life Technologies, ThermoFisher Scientific, Waltham, MA, USA). Transfections were changed to complete media after 4 h transfection, and cells were collected 48 h post transfection.

Viral particles were produced by HEK293T cells transfected with an ecopac packaging plasmid (PSPAX2 and PMD2G) (Addgene) and the retroviral PITA-SIRT1-FLAG expression plasmids. The supernatant carrying the viral particles was harvested after 48 h and concentrated 100 times. For viral transduction, EC cells (1 × 10^6^) were seeded in 2 mL DMEM supplemented with 10% FBS and 2 μl polybrene (8 mg/mL). EC cells were transduced with SIRT1 lentivirus at the infection MOI ≥ 90 for 24 h. After spinfection, the medium was changed into DMEM supplemented with 10% FBS, to remove polybrene and remaining viral particles. Cells were harvested after 48 h viral transduction.

### Immunoprecipitation

For immunoprecipitation, HEK293T cell lysates were precleared and incubated with 2 μg of capture antibody and 20 μl of 50% protein A/G-agarose bead slurry (Pierce Biotechnology, Rockford, IL, USA) overnight at 4 °C with gentle rotation. Immunoprecipitates were washed three times with modified RIPA buffer and boiled in 2 × Laemmli buffer, then subjected to SDS-PAGE electrophoresis and detection with specific antibodies against SIRT1, YAP, FLAG, or acetyl-lysine (AcK). The beads were washed. The specificity of antibodies used for immunoprecipitation was routinely validated by running negative controls using non-immune IgG using the same conditions as in formal experiments.

### Animal model

The experimental protocols described in this study were approved by Southwest Medical University of China. Female *ApoE*^−/−^ mice at 4–6 weeks of age were purchased from the SPF Biotech (Beijing, China) and maintained on a western chow diet for 12 weeks to generate vascular plaques. Randomized controlled trials (RCT) was used to blindly determine animal allocation to experimental groups. All experimental procedures were approved by the Department of Medical Ethics, Southwest Medical University. In all, 100 mg/kg diet rapamycin was added into the rodent diet, thoroughly mixed and then pellets were made. Subsets of mice from each group (*n* = 10) were weighed and euthanized after 8 weeks.

### Red-oil staining

Oil O Red staining of aortas was performed according to previous reference^15^. Briefly, aortas were stained with a freshly prepared Oil Red O working solution, differentiated by using 70% ethanol, mounted en face, and then observed by using a bright-field microscope (IX70, Olympus, Tokyo, Japan).

### Statistical analysis

Data are presented as mean ± standard error of the mean (SEM) for three biologically independent experiments if not explicitly stated otherwise. Data analysis was performed with unpaired *t*-test or one-way analysis of variance (ANOVA) followed by post hoc Tukey’s test as appropriate. A *P*-value of <0.05 was considered as statistically significant.
